# Intracranial neuro-enteric cyst: an illustrative patient and review

**DOI:** 10.11604/pamj.2020.37.26.20970

**Published:** 2020-09-07

**Authors:** Hajar Bechri, Mohamed Yassaad Oudrhiri, Meriem Fikri, Abdessamad El Ouahabi

**Affiliations:** 1 Neurosurgical Departement, Hôpital des Spécialités, Rabat, Morocco

**Keywords:** Neuroenteric, cyst, embryological remnants

## To the editors of the Pan African Medical Journal

**Figure 1:**
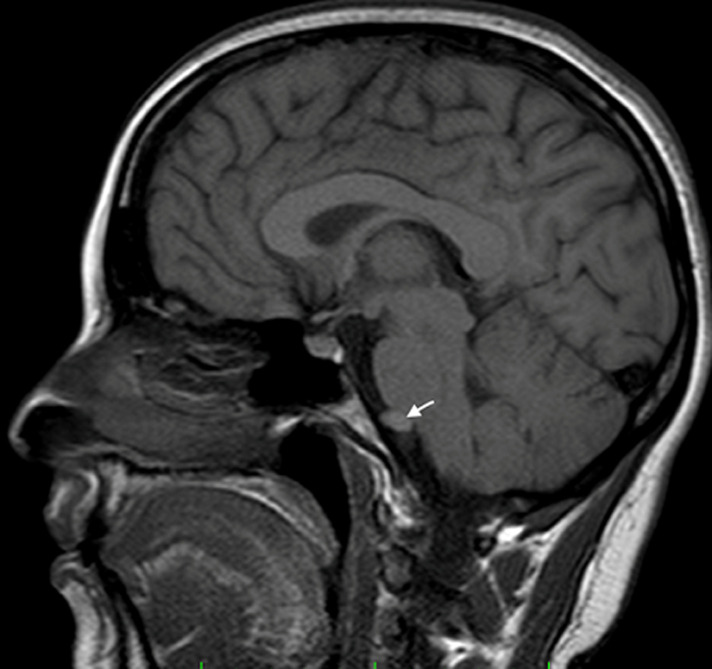
sagittal T1 MRI showing the isointense cystic extra axial lesion anterior to the brainstem

**Figure 2:**
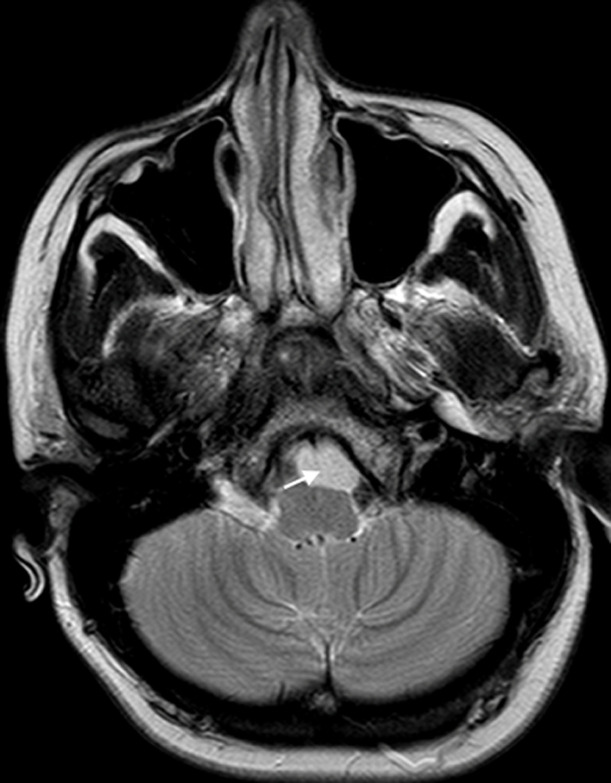
T2 axial MRI showing the lesion posterior to the clivus, with a brainsterm compression

28 years old female patient, with a history of gestational hypertension 6 months ago, presented with chronic headaches without symptoms of increased intracranial pressure. The neurological examination was normal, and the blood pressure was at 130/80 mmhg. A cranial CT scan was performed discovering a small isodense round lesion in the posterior fossa, in front of the brainstem. MRI scans confirmed an iso intense T1 hyper intense T2 extra-axial cystic lesion, with no contrast enhancement, and no diffusion restriction on DW images. It was located on the midline, anterior to the ponto-medullary junction, and seemed to have a cyst conduct through the inferior clivus (Figure 1and Figure 2). Considering the localization and the morphologic appearance, the lesion was diagnosed to be a neuro-enteric cyst (NEC). The poor clinical presentation, added to the size and location of the cyst made us propose an expectative attitude, and plan for a control MRI in 6 months that showed no increase of size of the lesion. Indeed, NEC are considered as embryological remnants. They are rare benign congenital lesions that can occur in any region of the central nervous system (CNS), predominantly in the spinal axis. Intracranial development is not unusual and mainly located in the posterior fossa, along the midline structures [1]. The etiology remains unclear; however, several hypotheses have been proposed. In fact, it has been discussed that NEC are consequent to the failure of involution of the neuroenteric canal between the primary neural tube and the enteric tube during the third and fourth week of gestation. Others have suggested adhesions causing abnormal development or duplication of the notochord [1].

On MRI imaging, they are typically round and/or lobulated, well margined extra-axial lesions [2,3]. The signal intensity may vary with the protein content of the cyst with a classical appearance of T1 hypo intensity, T2 hyper intensity, with no contrast enhancement and no diffusion restriction on DW images [3], making the differential diagnosis with the epidermoid cyst, arachnoid cyst and neurocysticercosis [2]. However, NEC may show atypical characteristics with mild diffusion restriction and wall cyst enhancement [2]. On microscopic examination, they are thin-walled with translucent structures. The content of the cyst varies from clear to mucoid or xanthochromic. The histopathological analysis of the wall shows endothelium lined structures of partially ciliated cuboidal to columnar cells. Pseudostratification can be observed in the epithelium at random intervals and characteristically has ciliated and goblet cells which makes them very resembling to the gastrinestinal tract [2]. Concerning the management, all authors agree on complete surgical excision in symptomatic patients [1,2]. Incomplete removal, usually due to the location of the cyst or the adherence to neural structures, leads to a recurrence rate up to 40% [1,2]. If not symptomatic, and because the majorities have been reported to be slow growing lesions, the monitoring by serial imaging is indicated [2].

## Conclusion

Neuroenteric cyst should be considered as a differential radiological diagnosis in the posterior fossa with the epidermoid cyst, arachnoid cyst and the neurocysticercosis. The treatment strategy depends on the clinical symptoms.

## References

[1] Gauden AJ, Khurana VG, Tsui AE, Kaye AH (2012). Intracranial neuroenteric cysts: a concise review including an illustrative patient. J Clin Neurosci.

[2] Nelson SM, Mathis DA, Hobbs JK, Timpone VM (2017). Intracranial neurenteric cyst mimicking an ependymoma: imaging features, pathologic correlation and review of literature. Clin Imaging.

[3] Singh P, Singh P, Aggarwal S, Tapasvi C (2017). Neurenteric Cyst: Magnetic Resonance Imaging Findings in an Adolescent. J Pediatr Neurosci.

